# Focal DNA Copy Number Changes in Neuroblastoma Target MYCN Regulated Genes

**DOI:** 10.1371/journal.pone.0052321

**Published:** 2013-01-04

**Authors:** Candy Kumps, Annelies Fieuw, Pieter Mestdagh, Björn Menten, Steve Lefever, Filip Pattyn, Sara De Brouwer, Tom Sante, Johannes Hubertus Schulte, Alexander Schramm, Nadine Van Roy, Tom Van Maerken, Rosa Noguera, Valérie Combaret, Christine Devalck, Frank Westermann, Geneviève Laureys, Angelika Eggert, Jo Vandesompele, Katleen De Preter, Frank Speleman

**Affiliations:** 1 Center for Medical Genetics, Ghent University Hospital, Ghent, Belgium; 2 Department of Pediatric Oncology and Haematology, University Children's Hospital Essen, Essen, Germany; 3 Department of Pathology, Medical School, University of Valencia, Valencia, Spain; 4 Centre Léon Bérard, FNCLCC, Laboratoire de Recherche Translationnelle, Lyon, France; 5 Children's University Hospital, Hematology-Oncology, Brussels, Belgium; 6 Department of Tumor Genetics, German Cancer Research Center, Heidelberg, Germany; 7 Department of Pediatric Hematology-Oncology, Ghent University Hospital, Ghent, Belgium; Institute of Cancerology Gustave Roussy, France

## Abstract

Neuroblastoma is an embryonic tumor arising from immature sympathetic nervous system cells. Recurrent genomic alterations include *MYCN* and *ALK* amplification as well as recurrent patterns of gains and losses of whole or large partial chromosome segments. A recent whole genome sequencing effort yielded no frequently recurring mutations in genes other than those affecting *ALK*. However, the study further stresses the importance of DNA copy number alterations in this disease, in particular for genes implicated in neuritogenesis. Here we provide additional evidence for the importance of focal DNA copy number gains and losses, which are predominantly observed in *MYCN* amplified tumors. A focal 5 kb gain encompassing the MYCN regulated *miR-17∼92* cluster as sole gene was detected in a neuroblastoma cell line and further analyses of the array CGH data set demonstrated enrichment for other MYCN target genes in focal gains and amplifications. Next we applied an integrated genomics analysis to prioritize MYCN down regulated genes mediated by MYCN driven miRNAs within regions of focal heterozygous or homozygous deletion. We identified *RGS5*, a negative regulator of G-protein signaling implicated in vascular normalization, invasion and metastasis, targeted by a focal homozygous deletion, as a new MYCN target gene, down regulated through MYCN activated miRNAs. In addition, we expand the miR-17∼92 regulatory network controlling TGFß signaling in neuroblastoma with the ring finger protein 11 encoding gene *RNF11*, which was previously shown to be targeted by the *miR-17∼92* member *miR-19b*. Taken together, our data indicate that focal DNA copy number imbalances in neuroblastoma (1) target genes that are implicated in MYCN signaling, possibly selected to reinforce MYCN oncogene addiction and (2) serve as a resource for identifying new molecular targets for treatment.

## Introduction

Neuroblastoma is an embryonal tumor arising from sympathetic neuronal progenitor cells and is responsible for 15% of all pediatric cancer deaths. This tumor exhibits a remarkably diverse clinical behavior ranging from spontaneous regression to aggressive disease, often refractory to multi-modal therapies [1]. Chromosomal and array comparative genomic hybridization (CGH) have been particularly instrumental in uncovering portraits of DNA copy number changes and allowed to establish a classification model consisting of three major genomic subtypes (termed subtype 1, 2A and 2B) predictive for clinical behavior [2]–[4]. Apart from the frequently occurring (large) DNA copy number alterations such as 17q gains and 1p, 3p and 11q deletions, the discoveries of rare focal genomic imbalances targeting *ALK* and *NF1* [5]–[10] and more recently also several genes implicated in neuritogenesis [11] have shown that such focal DNA copy number alterations mark important genes involved in neuroblastoma pathogenesis.

We recently identified the *miR-17∼92* cluster as an important mediator of MYCN signaling in neuroblastoma with several components of this cluster down regulating expression of target genes such as *DKK3* and members of the TGFβ pathway that contribute to the MYCN driven tumor phenotype [12]. Here we describe for the first time a focal 5 kb gain targeting exclusively the *miR-17∼92* polycistron in a neuroblastoma cell line. Further analyses of a large series of neuroblastoma tumors and cell lines (n = 223) revealed enrichment for known MYCN up regulated genes in focal gains and amplicons. Moreover, by applying an integrated genomics approach including mRNA and miRNA gene expression data and subsequent validation using *MYCN* and *miR-17∼92* inducible cellular model systems, we identified several genes, within the regions of focal loss, that are putatively under the control of MYCN activated miRNAs. Of these, *RGS5* and *RNF11* were identified as two new MYCN regulated therapeutically relevant genes, which are implicated in vascular remodeling [13], [14] and TGFß/NF-κß signaling [15]–[17], respectively.

## Results

### Increased miR-17∼92 copy number resulting from a focal 5 kb DNA copy number gain

**Figure 1:**
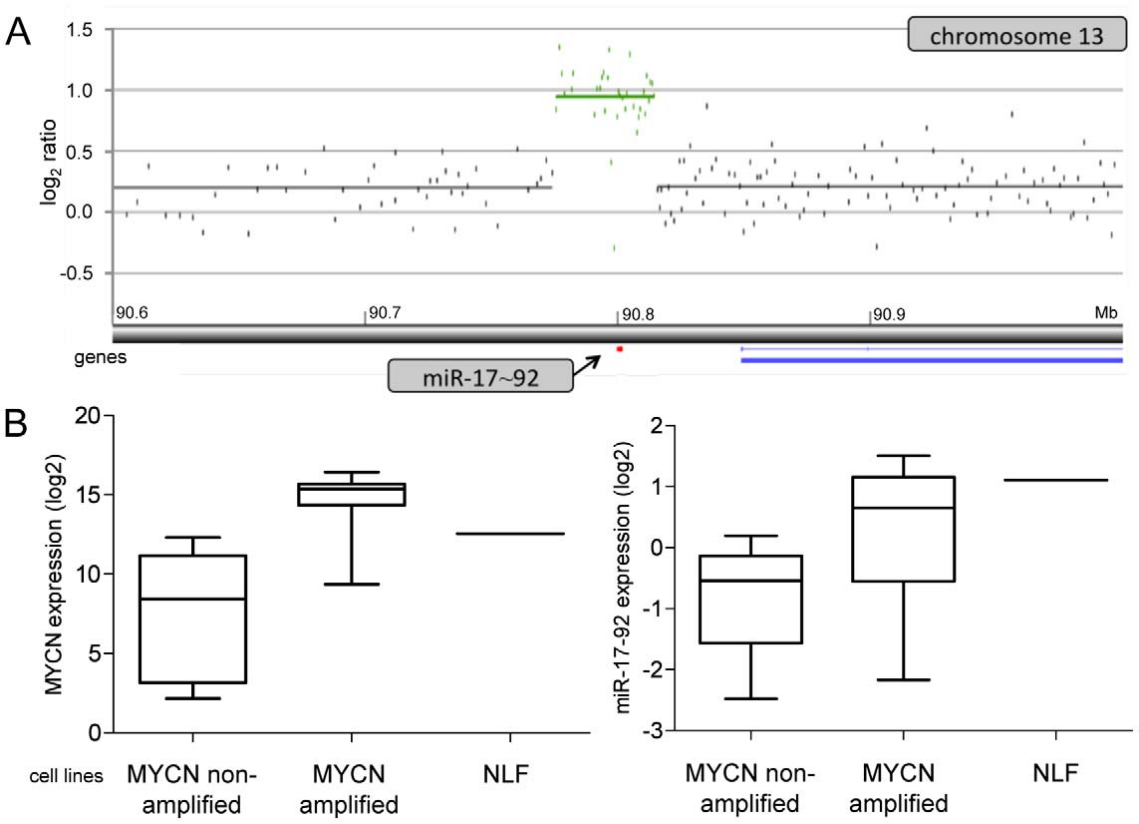
Focal gain of the MYCN activated miR-17∼92 cluster and expression levels in neuroblastoma cell lines. **A.** High resolution array CGH analysis shows a 5 kb focal gain for the *miR-17∼92* locus (13q31.3) in the *MYCN* amplified cell line NLF. **B.** Expression levels of *MYCN* (left) and the miR-17∼92 cluster (right) in 8 *MYCN* non-amplified and 18 *MYCN* amplified neuroblastoma cell lines as compared to the *MYCN* amplified NLF cell line.

High resolution array CGH analysis revealed, amongst others, a 5 kb focal gain of the *miR-17∼92* locus (13q31.3) in the *MYCN* amplified cell line NLF (Figure 1A). FISH analysis showed the presence of eight copies of the locus in this tetraploid cell line (data not shown). Expression levels of the *miR-17∼92* cluster were measured in 19 *MYCN* amplified and 8 *MYCN* non-amplified neuroblastoma cell lines. As expected, the *MYCN* amplified cell lines showed significantly increased miR-17∼92 levels in keeping with the previously described evidence for direct up regulation of the cluster by MYCN [18], [19]. MiR-17∼92 expression levels are not significantly higher in NLF cells as compared to the other *MYCN* amplified cell lines, but *MYCN* expression is within the lower range for NLF compared to the other *MYCN* amplified cell lines (Figure 1B), in keeping with relatively lower level of *MYCN* amplification (Figure S1). Therefore, we hypothesize that focal copy number gain of the *miR-17∼92* locus and consequent increased miR-17∼92 expression levels due to dosage effect may compensate for the relatively lower *MYCN* mRNA levels in cell line NLF. In accordance with this assumption, miR-17∼92 expression levels are indeed similar to the expression levels in *MYCN* amplified cell lines with higher MYCN expression levels. The observed focal gain of the *miR-17∼92* locus prompted us to further characterize focal DNA gains, amplifications and (homozygous) losses in a large series of primary neuroblastoma tumor samples and cell lines.

### Assessment of focal DNA copy number gains, amplifications and (homozygous) losses in primary neuroblastoma tumors and cell lines

A set of 33 neuroblastoma cell lines and 190 primary neuroblastoma tumors, representative of all clinical and genetic subtypes, was analyzed for focal DNA copy number aberrations (Table S1). In total, 14 homozygous, 4 hemizygous (located on chromosome X) deletions and 159 recurrent (present in at least 2 cases) focal (<2 Mb) heterozygous deletions as well as 118 amplifications and 22 recurrent focal gains were detected (Table S2) affecting 93 different chromosomal regions (see Materials and Methods for detailed description). Our analysis confirmed and further documented previously reported homozygous deletions involving several known tumor suppressor genes such as *KIF1B* (1p36.22), *CDKN2A/CDKN2B* (9p21.3) and *NF1* (17q11.2) as well as previously described focal heterozygous losses harboring genes such as *APAF1* (12q23), *ELAVL4* (1p34) and *PTPRD* (9p23-p24.3), while several amplifications were observed targeting well established oncogenes such as *MYCN* (2p24.1), *ALK* (2p23) and *MDM2* (12q14.3-q15) [9], [10], [20]–[23] (Table S2). Unbiased functional annotation analysis using the DAVID web tool [24] for genes located within regions of focal DNA copy number alteration revealed amongst others significant enrichment for pathways involved in “cell cycle and division” (including e.g. *CCND1*, *CDKN1C*, *CDK4*), “cellular apoptosis” (including e.g. *CDKN2A*, *DFFA*, *APAF1*) and “protein kinase” (including e.g. *INSR* and *NF1*). Given the implication of genes involved in activated tyrosine kinase pathways in neuroblastoma, *NF1* (a negative regulator of RAS signaling, targeted by homozygous loss), *INSR* and *ITK* (both tyrosine kinases, targeted by amplification) were selected for mutation analysis in a panel of neuroblastoma cell lines. Loss-of-function mutations in the *NF1* gene were detected in 23.5% of tested cell lines (confirming previous findings [10]) and a potentially damaging mutation in the *ITK* gene was detected in the tyrosine kinase domain in cell line NBL-S. No mutations were found for *INSR* (Table S1). Furthermore, we carefully compared data from our study with the complete genome sequencing data from Molenaar *et al.* (2012) [11] and found a significant overlap of regions affected by focal gains and losses including regions encompassing genes implicated in neuritogenesis or neuronal development (Table S3). Finally, when investigating the distribution of the focal gains and amplifications across the genetic subtypes, we observed that these focal aberrations occur more frequently in *MYCN* amplified samples (subtype 1, 2.6%; subtype 2A, 5.4%; subtype 2B, 48.8% (p_(FE (2B vs 1))_<1.0E-4, p_(FE (2B vs 2A))_<1.0E-4). Focal (homozygous) deletions are also observed more frequently in *MYCN* amplified samples, however this difference is not significant with subtype 2A (subtype 1, 22.4%; subtype 2A, 24.3%; subtype 2B, 41.9% (p_(FE (2B vs 1))_ = 3.6E-2; p_(FE (2B vs 2A))_ = 0.13) (Figure S2). When combining all focal aberrations, a significantly higher frequency was noted in subtype 2B tumors (p_(FE (2B vs 1))_<1.0E-4; p_((FE (2B vs 2A))_ = 1.9E-3).

### Focal DNA copy number gains are enriched for up regulated MYCN target genes

Further analysis showed that in addition to *miR-17∼92*, several known *bona fide* direct MYCN targets were affected by either gains or high level amplifications. A first example is the *TERT* gene [25] that was found for the first time as part of an amplified region at 5p15.33 in two MYCN non-amplified tumors (140 and 293 kb in size). In addition, a focal gain of this locus was found in one MYCN amplified primary tumor sample. DNA copy number analysis of these samples using qPCR revealed the presence of up to 41 *TERT* copies per haploid genome equivalent. Increased *TERT* expression could be confirmed in one amplified sample for which expression data were available, as compared to the tumors without *TERT* amplification (data not shown). Other examples of direct MYCN targets located within amplified regions in both our data set and previously reported array CGH data sets include *CDK4* (in one *MYCN* amplified and one *MYCN* non-amplified tumor and two *MYCN* amplified cell lines) and *MDM2* (in two *MYCN* amplified tumors and two *MYCN* amplified cell lines) [23], [26]. Of further interest, we observed several distinct regions on chromosome 2 that were co-amplified with *MYCN* and harbor direct MYCN target genes (i.e. *E2F6*, *ODC1* and *PPM1G*) [25], [27]. In particular, a region on chromosome 2 that is most often co-amplified with *MYCN* as a separate amplicon (in 4/43 *MYCN* amplified tumors (9.3%) and 2/24 MYCN amplified cell lines (8.3%)) contains the *bona fide* oncogene *ODC1* (2p25.1), which was recently described as a critical determinant of MYCN oncogenesis [28].

In a next step, we tested the regions of focal gain and amplification for enrichment of up regulated MYCN target genes, as available in the MYCNot database (http://medgen.ugent.be/MYCNot). A separate analysis was performed (1) for the up regulated target genes with a validated direct interaction with MYCN (referred to as directly up regulated MYCN target genes) and (2) for all up regulated genes in the MYCNot database, meaning all indirect MYCN targets, putative direct MYCN targets (for which currently no experimental proof is available) as well as the above mentioned validated direct MYCN target genes.

Investigation of focal gains and amplicons in our sample panel revealed significant enrichment for directly up regulated MYCN target genes in high risk MYCN non-amplified (subtype 2A) and MYCN amplified (subtype 2B) tumors as well as in the cell lines. When performing this analysis for all up regulated MYCN target genes listed in the MYCNot database, significant enrichment could be seen for all tumor subtypes and cell lines (Table 1; Table S3). Furthermore, we noticed that the regions containing direct MYCN targets in subtype 2A tumors i.e 5p15 (*TERT*) and 12q13.2-q14.1 (*CDK4*) are also gained or amplified in subtype 2B tumors (the indirect targets *INHBE*, *NACA* and *SHMT2*, also located in the 12q13.2-q14.1 amplicon were not found gained or amplified in the subtype 2B tumors).

**Table 1:** Enrichment analysis for (direct) MYCN target genes in focal aberrations in the clinico-genetic neuroblastoma tumor subgroups and cell lines.

p-value	all (n = 223)	1 (n = 76)	2A (n = 37)	2B (n = 43)	cell lines (n = 33)
**direct MYCN targets**					
**target up - gene gain**	0.087	1	0.012*	0.026*	9.5E-3*
**target down - gene deletion**	1	1	1	1	1
**all MYCN targets**					
**target up - gene gain**	1.81E-6*	0.031*	0.030*	4.12E-5*	3.28E-7*
**target down - gene deletion**	1	0.411	0.410	0.233	0.728

* significant p-value (p<0.05).

“all” includes also unclassified tumor samples (n = 34).

### Integrated genomic analysis for identification of genes down regulated by MYCN driven miRNAs and located within regions of focal genomic loss

Using the MYCNot database, no enrichment of MYCN down regulated target genes could be observed in the focal deletions. This was not unexpected, since so far only a limited number of down regulated MYCN target genes have been carefully assessed and validated. Recent studies revealed that MYCN-induced miRNA activation acts as an important mechanism for indirect down regulation of gene expression following MYCN overexpression [18], [19], [29]. We and others recently showed that *DKK3* and *ESR1* as well as several TGFβ pathway components were down regulated as a result of increased levels of miRNAs encoded by the *miR-17∼92* cluster in neuroblastoma [12], [30], [31]. Although for these genes no focal deletions were observed, we hypothesized that the deleted regions might encompass genes that are regulated by MYCN driven miRNAs suggesting an alternative mechanism to enforce MYCN downstream signaling, which thus far remained unstudied. To investigate this hypothesis, we used an integrative data mining approach that allows to pinpoint genes, located within regions of focal copy number loss, which are potentially negatively regulated by MYCN through MYCN driven miRNAs.

First, we investigated a cohort of 101 neuroblastoma samples for *MYCN* status and expression levels of MYCN activated miRNAs (as listed by Mestdagh *et al.* 2010 [18]) in relation to the mRNA expression level of each gene located within a focal loss. Candidate genes were defined as genes with expression levels significantly lower (p<5.0E-2) in MYCN amplified compared to MYCN non-amplified samples and that showed a significant negative correlation (p<5.0E-2) to the expression of at least one MYCN driven miRNA for which there was at least one 6-mer seed region present in the 3′UTR of the gene. Candidate genes were first ranked according to the lowest p-value representative for lower expression in MYCN amplified versus MYCN non- amplified samples and subsequently to the highest number of significant negative correlations to the expression of MYCN driven miRNAs (Table 2). This analysis generated a list of 38 candidate genes. Of interest, this selection contained genes present in 5 out of 10 regions of homozygous loss found in our study and several genes known to be implicated in cancer and neuronal differentiation (such as *NF1* and *GAP43*).

**Table 2:** Candidate genes located within regions of focal copy number loss and potentially down regulated by MYCN driven miRNAs.

chromosome	start deletion	end deletion	gene	t-test (MNA vs MNSC) lower in MNA	Nr of miRNAs with ≥7meR seeds and negative correlation	Nr of miRNAs with seeds and negative correlation	SHEP Tet21- N-MYC	SHEP-TR- miR-17-92
1	10085535	10521121	KIF1B*	2.57E-08	6	9	-	up
1	161412034	161439282	RGS5*	1.19E-05	5	7	down	down
1	50679369	52669864	RNF11	6.98E-08	5	5	down	down
9	8086071	8426577	PTPRD	8.54E-05	4	5	-	up
17	26309572	27044653	RAB11FIP4	4.98E-06	3	4	-	-
16	6368451	6894097	A2BP1	4.15E-05	3	4	-	-
1	10085535	10521121	UBE4B*	2.57E-05	2	4	-	-
1	36524504	36595081	STK40*	2.62E-03	2	3	down	up
1	50679369	52669864	EPS15	5.80E-05	1	4	NC	-
1	50679369	52669864	CC2D1B	2.97E-02	1	4	down	-
10	133846904	135202361	KNDC1	1.24E-06	1	3	-	-
3	116865083	117031992	GAP43	3.65E-05	1	3	-	down
1	50679369	52669864	RAB3B	6.90E-12	1	2	down	up
21	46734901	46905279	S100B	2.57E-06	1	2	-	NA
22	48241960	49525063	SAPS2	2.89E-05	1	2	down	-
22	21740181	22488949	GNAZ	1.93E-03	1	2	-	-
19	59746907	60384433	PTPRH	8.31E-08	1	1	down	-
21	46734901	46905279	PRMT2	1.08E-06	1	1	down	up
1	44215776	44216501	ATP6V0B	5.71E-06	1	1	-	-
18	74859078	75976412	ATP9B	6.30E-05	1	1	-	-
10	133846904	135202361	LRRC27	2.13E-03	1	1	-	NC
16	21517715	21645440	IGSF6*	4.08E-03	1	1	-	NA
17	26647443	26691593	NF1*	4.75E-03	1	1	-	-
7	69618669	69619814	AUTS2	8.50E-03	1	1	-	-
10	134446391	135192058	ECHS1	1.06E-02	1	1	-	-
2	112910493	113025949	TTL	1.66E-02	1	1	-	up
9	23691566	23751946	ELAVL2	4.68E-02	1	1	up	-
22	48241960	49525063	MAPK11	1.29E-02	0	2	-	-
17	26647443	26691593	EVI2A*	3.67E-02	0	2	-	NA
17	64797697	64802398	ABCA5	2.52E-06	0	1	down	-
18	33316912	33433533	BRUNOL4	3.21E-06	0	1	-	-
19	59746907	60384433	SYT5	1.99E-05	0	1	NA	-
10	134446391	135192058	INPP5A	1.98E-03	0	1	-	up
12	97512709	97566595	APAF1	4.89E-03	0	1	-	-
1	50690363	52669864	TXNDC12	8.13E-03	0	1	up	-
2	16597380	17715318	VSNL1	2.75E-02	0	1	-	-
11	2861519	2863443	CDKN1C	3.16E-02	0	1	-	down
22	21740181	22488949	RAB36	4.24E-02	0	1	-	-

* homozygous deletions.

NC: not conclusive.

NA: no data available.

In a next step, we sought further evidence for MYCN driven regulation of these candidate genes using the SHEP-TET21-N-MYC inducible model system [27], [32]. Nine candidate genes were significantly down regulated upon MYCN induction in this cell line marking these genes as potential candidates for down regulation by MYCN driven miRNAs. In order to find evidence for possible MYCN driven miRNA mediated down regulation, we further tested these genes in a second inducible model system for miR-17∼92 overexpression (SHEP-TR-miR-17∼92 model system; [12]). Upon miR-17∼92 induction, 4 out of 38 genes showed down regulation of which 2, *RGS5* and *RNF11*, were also down regulated after MYCN induction. Down regulation was confirmed for both genes by qPCR (Figure S3). Survival analysis in the same cohort of 101 neuroblastoma tumors showed association of decreased mRNA expression levels of both *RGS5* and *RNF11* with poor survival (p = 4.8E-2; p = 6.4E-4), which is in line with the observed association of both genes with *MYCN* expression. *RNF11* was recently shown to be regulated by the miR-19b component of the miR-17∼92 cluster [15] in keeping with the above findings.

### The homozygously deleted RGS5 gene is a newly identified indirect MYCN down regulated gene

**Figure 2:**
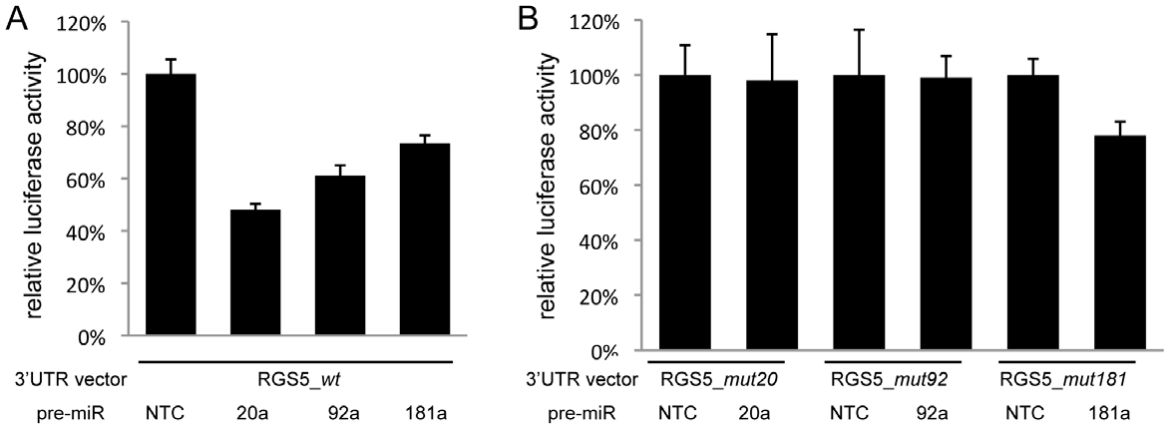
miRNA interaction with the 3′UTR of RGS5. **A.** Relative luciferase activity obtained by transfecting cells with the wild type 3′UTR of RGS5 in combination with pre-miR-negative control (NTC), pre-miR-20a, pre-miR-92a and pre-miR-181a in HEK293T cells. **B.** Relative luciferase activity obtained by transfecting cells with mutated 3′UTR of RGS5 in combination with pre-miR-NTC, pre-miR-20a, pre-miR-92a and pre-miR-181a in HEK293T cells.

*RGS5* is homozygously deleted on chromosome 1q23 in cell line NLF. Using ultra-high density (1M) oligo array CGH breakpoints of this 27 kb deletion were further delineated and complete loss of RGS5 expression was confirmed by qPCR (Figure S4). In a next step, we further investigated the presumed regulation of RGS5 by the MYCN induced miR-17∼92 cluster. There was no evidence for direct down regulation of RGS5 by MYCN based on MYCN chromatin immunoprecipitation (ChIP) sequencing data of four neuroblastoma cell lines (data not shown). Furthermore, given the presence of multiple seed regions of MYCN driven miRNAs in the *RGS5* 3′UTR and significant negative correlation between *RGS5* expression and seven of these miRNAs (Table S4), we evaluated their repressive effect on *RGS5* expression. We selected four of the seven MYCN driven miRNAs (p<5.0E-2): miR-9, miR-20a, miR-92a and miR-181a and tested binding to the 3′UTR of RGS5. To this purpose, HEK-293T cells were co-transfected with a 3′UTR luciferase reporter plasmid, containing the full length 5074 bp *RGS5* 3′UTR, and the selected pre-miRs. This showed significant decrease in luciferase activity for miR-20a, miR-92a and miR-181a indicating a direct interaction between these miRNAs and the 3′UTR of *RGS5* (Figure 2A). No evidence for binding was obtained for miR-9 thereby excluding 3′UTR-mediated direct regulation of *RGS5* expression for this miRNA. Mutation of the active 7mer miRNA seed regions for miR-92a and two 6mer seeds for miR-20a allowed to rescue the luciferase output in keeping with the assumption that the effect on luciferase reduction depends on the presence of the active miRNA seed sequences in the 3′UTR of *RGS5* (Figure 2B). A rescue effect after mutation of the 7mer seed for miR-181 could not be observed. *RGS5* mRNA down regulation following miR-17∼92 induction in the SHEP neuroblastoma cell line (SHEP-TR-miR-17∼92; [12]) is in keeping with the above described direct interaction between two members of the *miR-17∼92* cluster, i.e. *miR-20a* and *miR-92a* and the 3′UTR of *RGS5* (fold change = 1.53).

In addition to posttranscriptional down regulation, we also investigated the possibility that RGS5 might be targeted by loss-of-function mutations but sequencing of a panel of cell lines revealed no mutations, in keeping with low mutation rate in neuroblastoma [11], which was published following our RGS5 sequencing analysis.

## Discussion

**Figure 3:**
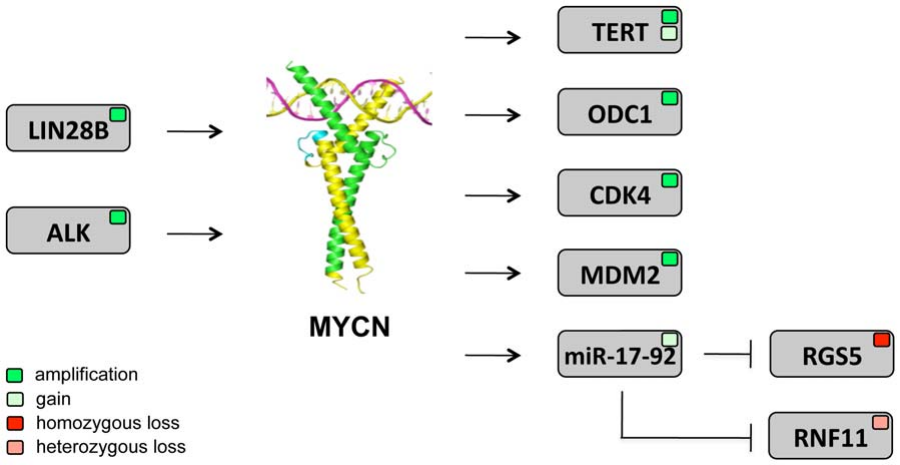
Rare recurrent focal copy number changes in neuroblastoma target up and downstream MYCN pathway components. Illustration of genes in focal genomic gains (light green) and amplifications (dark green) that are direct up regulated MYCN target genes or upstream regulators of MYCN and genes in focal heterozygous (light red) and homozygous (dark red) deletions which are down regulated by MYCN through its activated miRNAs. Protein structure was obtained from www.pdbe.org.

MYCN is a crucial driver gene in the childhood cancer neuroblastoma as illustrated by overexpression due to genomic amplification in a large subset of high risk tumors and formation of neuroblastoma in MYCN-driven transgenic mouse and zebrafish model systems [33]–[37]. The recently demonstrated functional and synergistic relationship between MYCN and ALK, another important oncogene in neuroblastoma, further reinforces the central role of MYCN in neuroblastoma oncogenesis [35], [38]–[40]. With the exception of ALK, for which activating mutations are observed in 7% of tumors, single nucleotide substitutions are rarely observed in neuroblastoma [11]. Thus far, the genomic landscape of neuroblastoma seems to be dominated by DNA copy number alterations and chromothripsis, for the latter the functional consequence remains to be established. Combined analysis of rare mutations and focal DNA copy number changes provided evidence for their functional relevance to the tumor cell given the implications of many of these genes in the control of normal neuritogenesis [11]. Here, we add further evidence for selective advantage for tumor cells exhibiting focal gains and losses encompassing genes under direct or indirect transcriptional control of MYCN (Figure 3). First, we observed in cell line NLF a focal gain of *miR-17∼92*, an important oncogenic locus under the direct transcriptional control of MYCN. In this cell line, *MYCN* is amplified but with moderate increased copy number levels. In keeping with this observation, *MYCN* mRNA expression is clearly elevated but within the lower range of the cohort of *MYCN* amplified cell lines. We therefore hypothesize that *miR-17∼92* copy number gain may have been selected to reinforce the MYCN-driven oncogenic phenotype in this cell line as this miRNA cluster is a well-established direct oncogenic target of MYCN and MYC and is consistently up regulated in *MYCN/MYC* amplified neuroblastoma as well as in other tumor entities [18], [41]. Further investigation of a large array CGH data set of neuroblastomas confirmed several previously reported rare recurrent amplicons. These regions contain *bona fide* up regulated MYCN target genes such as *ODC1*, *TERT*, *CDK4* and *MDM2* (Figure 3) [23], [25], [26], [28]. Using the MYCNot database, we found enrichment of both directly and indirectly up regulated MYCN target genes in the focal DNA copy number gains. Encouraged by these findings, we also explored the genes that were targeted by focal deletions. Using an integrated genomics approach by combining DNA copy number data, mRNA and miRNA gene expression data as well as miRNA seed information, we selected 38 candidate genes for down regulation by MYCN activated miRNAs. Using a MYCN and miR-17∼92 inducible cell line model system, we identified two new genes regulated by components of the MYCN regulated miR-17∼92 cluster, i.e. *RGS5* and *RNF11* (Figure 3). *RGS5* encodes for a negative regulator of G-protein signaling implicated in vascular normalization, invasion and metastasis and its involvement was shown in breast and lung cancer. More specifically, reduced expression of RGS5 was associated with increased tumor aggressiveness and poor survival in keeping with our observations in NB [13], [14], [42], [43]. The *in vivo* role of RGS5 in neuroblastoma tumor vascularity is currently under further investigation.

*RNF11* is an important regulator of TGFβ, EGFR and NF-κβ signaling and mediates the ubiquitination and proteolysis of many cellular proteins [15], [17], [44]. The role of RNF11 in the TGFβ pathway is of particular interest in neuroblastoma given our previous finding that the miR-17∼92 cluster components target *TGFBR2* and *SMAD2* as well as TGFβ downstream regulated genes *CDKN1A*, *ITGA4*, and *SERPINE1* [12]. RNF11 enhances TGFβ signaling through rescue of SMAD2 from degradation and by blocking the activity of SMURF2, the latter that interacts with inhibitory SMADs to target the TGFβ receptor for destruction via proteasomal degradation. RNF11 can also bind directly to SMAD4 and directly regulate SMAD4 transcriptional activity [16], [17]. It should be noted that SMURF2 is located on 17q24.1, one of the most frequently affected regions by partial and whole chromosome gains in neuroblastoma. Further studies are necessary to explore the possible interaction between MYCN/MYC up regulation and *SMURF2* copy number gains in neuroblastoma in relation to suppression of TGFβ signaling.

The present study investigates gene content of focal gains, amplicons and deletions in neuroblastoma. Interestingly, these focal aberrations occur more frequently in *MYCN* amplified tumors (this study) whereas a higher frequency of large segmental aberrations was observed in subtype 2A tumors in keeping with previously published data [45]. Most of the more frequent gains and amplifications contain known *bona fide* oncogenes such as *ODC1* (n = 7; 3.1%) [28], *CDK4* (n = 4; 1.8%) [46], [47], *MDM2* (n = 4; 1.8%) [48], [49] and *ALK* (n = 4; 1.8%) [39], [50]. Several of the more frequent deletions (including homozygous ones) harbor known tumor suppressor genes such as *CDKN2A* (n = 4; 1.8%) [51], [52], *NF1* (n = 5; 2.2%) [10], *PTPRD* (n = 5; 2.2%) [53], [54] and genes involved in apoptosis such as *APAF1* (n = 4; 1.8%) [55], *IKIP* (n = 4; 1.8%) [56], *WWOX* (n = 4; 1.8%) [57], [58] and *FAF1* (n = 4) [59]. Importantly, this study provides further evidence that (recurrent) focal genomic alterations contribute to the cancer cell phenotype through reinforcing the effects of multiple components of a single pathway as recently illustrated for the p53 and cell cycle pathway in diffuse large B cell lymphoma [60] and for the NOTCH pathway in T-ALL [61]. More specifically, we show here that a significant proportion of such focal copy number changes target genes implicated in MYCN signaling and may therefore reinforce the oncogenic effect of MYCN (or MYC) on neuroblastoma cells. Interestingly, this observation is also in line with other genomic alterations targeting upstream regulators of MYCN activity (Figure 3). Recently, mutant and wild type ALK was shown to directly regulate MYCN transcription levels [40], [62] and increase MYCN protein stability through PI3K/AKT driven phosphorylation of S62 [63], [64]. Of further notice, LIN28B, a negative regulator of the let-7 family of miRNAs targeting *MYCN* mRNA, was amplified in rare neuroblastoma cases and more recently transgenic mice overexpressing *LIN28B* in the developing sympathetic nervous tissue were shown to develop neuroblastoma [65]–[67]. Therefore, a picture is emerging of a darwinistic genetic wiring in neuroblastoma cells in which multiple events strive to increase MYCN downstream activity.

## Materials and Methods

### Tumor samples and cell lines

A series of 190 primary neuroblastoma tumor samples was collected prior to therapy (56 cases from Ghent University Hospital, Belgium, 33 from the Medical School of Valencia, Spain and 101 from Essen University Children's Hospital, Germany) for which a written informed consent was obtained and approved by the commision for medical ethics of the respective institutions (#08-3670; EC/2006-146/Svdm; EC/2007-137/Svdm; 2010/037). In addition, 33 neuroblastoma cell lines were included in this study. Patient and cell line information (including cell line references) are summarized in Table S1. DNA was isolated from primary tumors and cell lines using the Qiagen DNA isolation kit (Qiagen, Cat nr 51304) according to the manufacturer's instructions. Total RNA was isolated using the miRNeasy kit (Qiagen) according to the manufacturer's instructions including DNase treatment on column. miRNA and mRNA expression data of 101 tumors was previously published [12], [18] and data is available at the Gene Expression Omnibus (GEO, http://www.ncbi.nlm.nih.gov/geo/ accession number: GSE32664).

### Array Comparative Genomic Hybridization (array CGH) and data analysis

Samples were profiled on a custom designed 44K (156 samples), 60K (66 samples) or 180K (1 sample) array (Agilent Technologies) enriched for critical regions in neuroblastoma (1p, 2p, 3p, 11q, 17), cancer gene census genes [68], non-coding sequences (miRNAs and transcribed ultra-conserved regions) and all genes from the Neuroblastoma Gene Server (NBGS) database [69]. The latter database contains curated lists of differentially expressed genes retrieved from all major published high-throughput neuroblastoma gene expression profiling studies. Validation experiments were performed on the commercially available 1M array (Agilent Technologies). The array designs are based on the hg18 genome build. Utilizing random prime labeling (BioPrime ArrayCGH Genomic Labeling System, Invitrogen), 400 ng (44K/180K), 200 ng (60K) or 1 µg (1M for validation purposes) of tumor and control DNA was labeled with Cy3 and Cy5 dyes (Perkin Elmer), respectively. Further processing was then performed according to the manufacturer's instructions (Agilent Technologies). Fluorescence intensities were measured using an Agilent scanner (G2505C, Agilent Technologies). Data were extracted using the Feature Extraction v10.1.1.1 software program (Agilent Technologies) and further processed with arrayCGHbase (http://medgen.ugent.be/arraycghbase) [70]. Array CGH profiles were further processed using the “no waves” [71] and the circular binary segmentation (CBS) algorithm [72] that splits the genomic data into contiguous regions of equal copy number, and uses permutation to define copy number abnormalities at a defined significance level. The statistical environment R (version 2.10.0) was used to identify the location of all possible amplifications, homozygous deletions and recurrent focal gains and losses, whereas gene information was extracted using BioMart [73] and then used to determine the genes residing within these regions. The miRBase database was used to extract the chromosomal locations of the miRNAs and to identify in which regions these reside. The following criteria were used to select copy number alterations (CNA) inferred by DNA copy number analysis for further validation and study: (a) homozygous deletion (log2_(CBS value)_<−2.0), (b) amplification (log2_(CBS value)_>2.0), (c) focal recurrent loss and (d) focal recurrent gain. We defined focal losses and gains for (c) and (d) as alterations <2 Mb involving at least 2 non-overlapping contiguously altered oligos (log2_(CBS value)_<−0.3 and >1.0, respectively). Since we aimed to find genes important in neuroblastoma biology we looked for recurrent alterations defined by their occurrence in at least 2 samples. Chromosomal regions that are homozygously deleted or amplified (>8 haploid copies) are typically small and very likely to contain tumor suppressor genes and oncogenes, respectively. Therefore, all (and not only recurrent) homozygously deleted regions or amplicons were taken into account for further analysis. Throughout the text we will refer to (a) and (c) as focal copy number losses and to (b) and (d) as focal copy number gains. The Y chromosome was excluded from the analysis. Copy number variants characteristic for control DNAs were excluded. Aberrations that were present in more than 5 cases in a separate in-house screened clinical genetics cohort of 1000 samples were considered as possible copy number variants and excluded from further analysis (http://medgen.ugent.be/arraycghbase). All reported results were confirmed by manual inspection of the array profiles. All homozygous deletions, amplifications and focal recurrent losses and gains that met the aforementioned criteria are provided in Table S2. Thirty-eight regions were tested for validation using FISH, qPCR or ultra-high resolution oligonucleotide arrays and aberrations could be confirmed for 33 regions (87%) (Table S2). All array data are available at http://medgen.ugent.be/arraycghbase (login: guest, password: guest).

### MYCN oncogene targets (MYCNot) database

To summarize the current knowledge on MYCN downstream cascades, a thorough literature search was performed to track down all hitherto identified MYCN downstream genes. We grouped all the information and assembled and annotated the individual genes into the ‘MYCN oncogene targets’ (MYCNot) database (http://medgen.ugent.be/MYCNot). Most of the genes listed in the database have been identified as putative MYCN target genes through one or more differential expression screens without any other evidence suggesting that they are directly regulated by MYCN alone or by both MYCN and other transcription factors that work in a co-ordinated manner. Currently, 1041 up and down regulated MYCN mRNA targets are included in this database.

### Real-Time quantitative Polymerase Chain Reaction (qPCR)

Real-Time qPCR was performed for validation of DNA CNAs and for expression evaluation. DNA and RNA concentration was measured using the Nanodrop (Thermo Scientific). First-strand cDNA was synthesized from total RNA using an iScript cDNA synthesis kit (Bio-Rad). qPCR reactions were performed with SYBR Green I detection chemistry, using the LC480 real-time PCR detection system (Roche). qPCR reactions were performed in a total volume of 5 µl consisting of 2.5 µl of SYBR Green I qPCR master mix (2×) (Eurogentec, HotGoldStar polymerase), 0.25 µl of forward and reverse primer (5 µM), 2 µl of 2.5 ng/µl DNA or cDNA (total RNA equivalents). Cycling conditions were as follows: 10 minutes at 95°C followed by 45 cycles of 10 seconds at 95°C, 45 seconds at 60°C and 1 second at 72°C. DNA copy number levels were measured relative to human genomic DNA (Roche Diagnostics) and normalized to the internal reference genes, *BMCA* (ID 14) and *SDC4* (ID 15). mRNA expression levels were measured and normalized to at least 2 internal reference genes, *TBP* (ID 8098), *YWHAZ* (ID 8100), *B2M* (ID 2) and *UBC* (ID 8099). Primer sequences are available in RTPrimerDB (http://www.rtprimerdb.org) [74], ie *hsa-miR26a-2* (ID 8243), *hsa-miR-766* (ID 8250), *RGS5* (ID 8244), *TCF4* (ID 8245), *ITK* (ID 8246), *INSR* (ID 8247), *TERT* (ID 8248), *SEPT6* (ID 8249) *CDKN2A* (ID 8251), *NF1* (ID 8252), *DIAPH2* (ID 8254), *IGSF6* (ID 8255), *THRAP3* (ID 8257), *A2BP1* (ID 8258), *MYCN* (ID 11), *RGS5* (ID 8261-expression) and RNF11 (ID 8592-expression). Copy number and mRNA data analysis as well as error propagation was done using qbase^PLUS^ software 1.5 (http://www.biogazelle.com) [75]. All real-time qPCR assays were conducted in duplicate.

### Expression analysis of neuroblastoma cellular model systems and cell lines

SHEP-TET21N-MYC cells were cultured in RPMI (Invitrogen) supplemented with 10% fetal calf serum and treated with 1 µg/ml tetracycline (Sigma-Aldrich) to switch off MYCN expression (as reported in [32]). Publically available expression data from tetracycline treated and untreated SHEP-TET21N-MYC cells [32] were downloaded from the ArrayExpress website (accession number E-MEXP-2340) and validated for selected markers using qPCR. SHEP-TR-miR-17∼92 cells were cultured in RPMI (Invitrogen) supplemented with 10% fetal calf serum and treated with 2 µg/ml tetracycline (Sigma-Aldrich) to induce miR-17∼92 expression as reported in [18]. Expression data from the tetracycline treated and untreated miR-17∼92 inducible neuroblastoma SHEP cell line system were analysed using Affymetrix GeneChip Human Gene 1.0 ST arrays [18] (Table S5). Fold changes larger than 1.2 were considered potentially relevant in this model system and were subsequently validated using qPCR. MYCN and miR-*17∼92* expression level were determined for 27 neuroblastoma cell lines for which gene expression profiling was performed using Sureprint G3 human gene expression microarrays (Agilent Technologies) according to the manufacturer's instructions as well as miRNA profiling using a stemloop RT-qPCR platform as described previously [76], [77] (Table S6).

### Statistics

Statistical analyses were performed with the statistical environment R. Enrichment analyses were done using a Fisher exact (FE) test. For the prioritization of genes in focal losses, selection of the candidate genes was performed by combining data of spearman correlation analysis between miRNA and mRNA expression data and paired t-test. Statistical significance was defined as p<5.0E-2.

### Sequencing

Sequence analysis of the coding exons and flanking intronic sequences of *RGS5* and the tyrosine kinase domain of *ITK* was performed on genomic DNA. *NF1* (using emetin treated material [78]) and the tyrosine kinase domain of *INSR* were sequenced on the cDNA level. PCR products were subjected to unidirectional sequencing using BigDye Terminator V1.1/V3.1 Cycle Sequencing chemistry using an ABI3730XL sequencer (Applied Biosystems). Electropherograms were analyzed using Seqscape v2.5 software (Applied Biosystems). Primer info is summarized in Table S7.

### 3′UTR luciferase assay

The 3′UTR of the human *RGS5* gene was cloned in a vector downstream of the firefly luciferase open reading frame (pMirTarget vector, Origene). Seed regions were mutated using the QuickChange II mutagenesis kit (Stratagene). Specifically, the complementary sites of miR-20a (2), miR-92a and miR-181a, CCACACATACACACACA**C**A**CTT**TTTGTTTCTTTCAGGTAGAC and CTGTTTGTGTTAAACA**C**A**CTT**TTCACCAAATAGGTTC, TATTCACATTATT**T**G**CAA**TATCCAAATGTTTAAAAATTC and GACTATGATATCAA**T**G**AAT**GTGGGTTAAGTAATAG were changed into CCACACATACACACACA**A**A**AGG**TTTGTTTCTTTCAGGTAGAC and CTGTTTGTGTTAAACA**A**A**AGG**TTCACCAAATAGGTTC, TATTCACATTATT**G**G**ACC**TATCCAAATGTTTAAAAATTC and GACTATGATATCAA**G**G**CCG**GTGGGTTAAGTAATAG, respectively. HEK-293T cells were plated at 10,000 cells per 96-well in RPMI medium containing 10% FBS 24 hrs prior to transfection. Transfection mixes contained RPMI medium, 50 nM pre-miR (Applied Biosystems), 0.4% DharmaFect Duo (Dharmacon) and 100 ng oligo-psiCHECK vector. The ratios between Firefly and Renilla luciferase were measured 48 hr post-transfection using the dual-glo luciferase kit (Promega) according to the manufacturer's protocol.

## Supporting Information

Figure S1 ***MYCN*** **copy number and gene expression for neuroblastoma cell lines.** *MYCN* copy number data (log_2_) and *MYCN* expression data were plotted for a panel of *MYCN* amplified and *MYCN* non-amplified cell lines. *MYCN* expression levels were significantly higher in the *MYCN* amplified cell lines (**; p_(Mann-Whitney)_<0.001). A linear relation was observed between *MYCN* copy number and expression within the group of *MYCN* amplified cell lines (p<0.005).
(TIF)

Figure S2 **Distribution of focal aberrations.** A. Distribution of recurrent focal gains and amplifications and B. recurrent focal losses and homozygous deletions in the neuroblastoma tumors according to subtype. Amplification of the MYCN locus was excluded for this analysis as MYCN amplification is characteristic of subgroup 2B. ***, p_(FE)_<0.0001; *, p_(FE)_<0.05
(TIF)

Figure S3 **Confirmation of** ***RGS5*** **and** ***RNF11*** **down regulation.** A. Confirmation of *RGS5* and *RNF11* down regulation using qPCR after induction of MYCN in the SHEP-TET21-N-MYC and B. miR-17∼92 in the SHEP-TR-miR-17∼92 inducible model systems.
(TIF)

Figure S4 **Confirmation of** ***RGS5*** **homozygous deletion in NLF.** A. Confirmation of *RGS5* homozygous deletion by 1M high-resolution array CGH. B. *RGS5* mRNA expression by qPCR in NLF versus average expression in NB cell lines.
(TIF)

Table S1 **Overview of patient and cell line information.**
(XLSX)

Table S2 **Overview of focal aberrations in tumors and cell lines.**
(XLSX)

Table S3 **Detail of enrichment analyses.**
(XLSX)

Table S4 **Correlation of MYCN regulated miRNAs to** ***RGS5*** **and** ***RNF11*** **expression and miRNA seed information.**
(XLSX)

Table S5 **Fold change data from the tetracycline treated and untreated SHEP-TR-miR-17∼92 inducible model using Affymetrix GeneChip Human Gene 1.0 ST arrays** [**18**] **.**
(XLSX)

Table S6 ***MYCN*** **amplification status and expression and miR-17∼92 expression in a panel of neuroblastoma cell lines.**
(XLSX)

Table S7 **List of sequencing primers.**
(XLSX)
