# Supplementary figures and images for: Focal DNA Copy Number Changes in Neuroblastoma Target MYCN Regulated Genes
Source: PLoS One. 2013 Jan 4;8(1):e52321. doi: 10.1371/journal.pone.0052321 (PMC3537730; doi:10.1371/journal.pone.0052321)

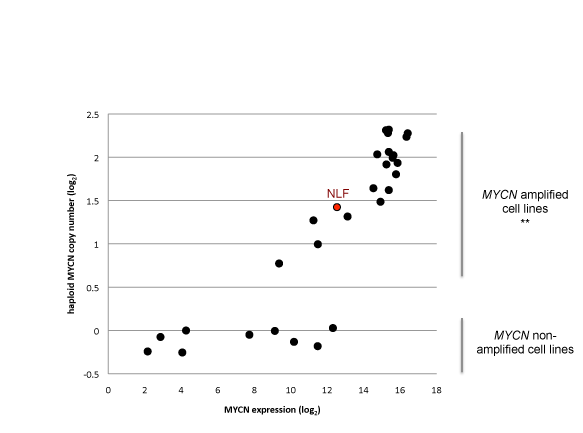

Supplement: Figure S1 — MYCN copy number and gene expression for neuroblastoma cell lines. MYCN copy number data (log2) and MYCN expression data were plotted for a panel of MYCN amplified and MYCN non-amplified cell lines. MYCN expression levels were significantly higher in the MYCN amplified cell lines (**; p(Mann-Whitney)<0.001). A linear relation was observed between MYCN copy number and expression within the group of MYCN amplified cell lines (p<0.005). (TIF) [file pone.0052321.s001.tif]

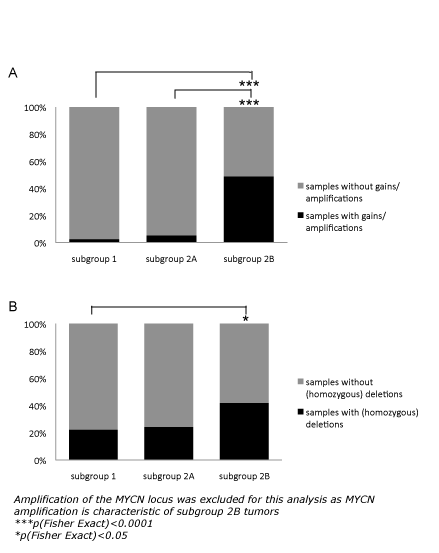

Supplement: Figure S2 — Distribution of focal aberrations. A. Distribution of recurrent focal gains and amplifications and B. recurrent focal losses and homozygous deletions in the neuroblastoma tumors according to subtype. Amplification of the MYCN locus was excluded for this analysis as MYCN amplification is characteristic of subgroup 2B. ***, p(FE)<0.0001; *, p(FE)<0.05 (TIF) [file pone.0052321.s002.tif]

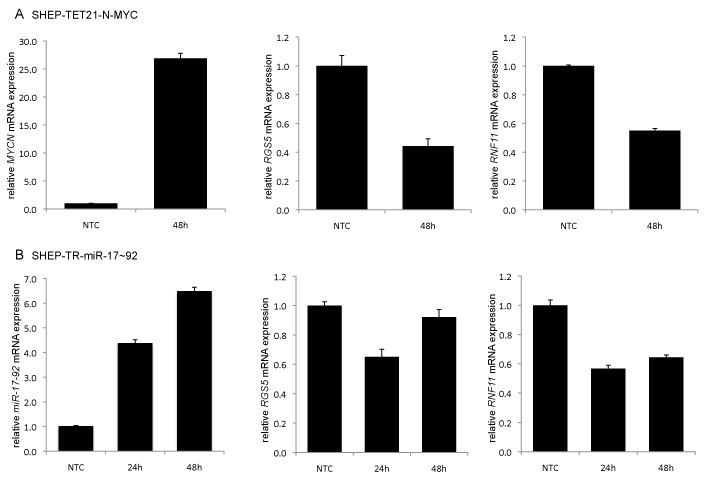

Supplement: Figure S3 — Confirmation of RGS5 and RNF11 down regulation. A. Confirmation of RGS5 and RNF11 down regulation using qPCR after induction of MYCN in the SHEP-TET21-N-MYC and B. miR-17∼92 in the SHEP-TR-miR-17∼92 inducible model systems. (TIF) [file pone.0052321.s003.tif]

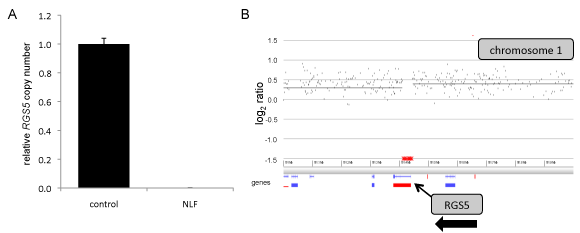

Supplement: Figure S4 — Confirmation of RGS5 homozygous deletion in NLF. A. Confirmation of RGS5 homozygous deletion by 1M high-resolution array CGH. B. RGS5 mRNA expression by qPCR in NLF versus average expression in NB cell lines. (TIF) [file pone.0052321.s004.tif]
